# Nadir creatinine as a predictor of renal outcomes in PUVs: A systematic review and meta-analysis

**DOI:** 10.3389/fped.2023.1085143

**Published:** 2023-03-15

**Authors:** Davide Meneghesso, Nicola Bertazza Partigiani, Rachele Spagnol, Alessandra Rosalba Brazzale, Alessandro Morlacco, Enrico Vidal

**Affiliations:** ^1^ Pediatric Nephrology, Department of Womens’s and Children's Health, University Hospital of Padua, Padua, Italy; ^2^ Department of Statistical Sciences, University of Padua, Padua, Italy; ^3^ Pediatric Urology Unit, Padua University Hospital—Department of Surgical, Oncological and Gastroenterological Sciences, Padua University, Padua, Italy; ^4^ Department of Medicine (DAME), University of Udine, Udine, Italy

**Keywords:** nadir creatinine, chronic kidney disease, posterior urethral valves, predictive factor, posterior urethral valve (PUV)

## Abstract

**Background:**

Posterior urethral valves (PUVs) represent the most severe pediatric obstructive uropathy, responsible for chronic renal failure in up to 65% of cases and progression to end-stage kidney disease (ESKD) in about 8%–21% of patients. Unfortunately, renal outcomes have poorly improved over time. The key point is to identify patients at risk; thus, several prenatal and postnatal prognostic factors have been analyzed to improve clinical outcomes. Postnatal nadir creatinine seems to accurately predict long-term renal prognosis, but there is no definitive evidence to support this finding.

**Objective:**

We performed a systematic review with meta-analysis to analyze the predictive value of nadir creatinine on long-term renal function in infants with PUVs.

**Methods:**

We conducted this systematic review according to the Preferred Reporting Items for Systematic Reviews and Meta-Analyses (PRISMA) guidelines. PubMed and Cochrane Library were systematically searched for studies published from January 2008 to June 2022. All the articles were checked independently by two reviewers in two steps.

**Results:**

A total of 24 articles were screened, and 13 were included for data extraction. Data from 1,731 patients with PUVs were analyzed, with a mean follow-up of 5.5 years; of these, on average, 37.9% developed chronic kidney disease (CKD) and 13.6% developed ESKD. All the articles evaluated nadir creatinine as a predictor of CKD, most using a level of 1 mg/dL, with statistical significance at the 5% level. The relative risk of developing CKD in patients with creatinine values higher than the nadir cutoff considered was 7.69 (95% CI: 2.35–25.17, *I*^2^ = 92.20%, *p* < 0.001).

**Conclusions:**

Nadir creatinine is the best prognostic factor for long-term renal function in patients affected by PUV. A value above the cutoff of 1 mg/dL should be considered a significant predictor for the risk of CKD and ESKD. Further studies are needed to define different nadir creatinine cutoffs for better stratification of the different CKD stages and for the development of reliable scores, which include the association of several variables.

## Background

Posterior urethral valves (PUVs) represent the most common cause of lower urinary tract obstruction (LUTO) in males with bilateral hydronephrosis (1, 2). PUVs can cause a decrease in fetal urinary output resulting in oligohydramnios with secondary pulmonary hypoplasia in the prenatal period, which is a major cause of perinatal death and associated with early mortality and morbidity by causing disorders of renal development (3–5). PUVs appear as intraluminal folds located immediately proximal to the verumontanum causing partial or complete obstruction to the urinary outflow. According to recent literature, infants with PUV may develop chronic kidney disease (CKD) in 20%–65% and progress to end-stage kidney disease (ESKD) in about 8%–21% of patients (6–8). Endoscopic resection of PUVs is the initial treatment in most patients; more rarely, an early temporary urinary diversion is required (9–12). Despite improving surgical techniques and early diagnosis, the renal outcome has poorly improved over time (13–15). Many prognostic factors have been analyzed both in prenatal and postnatal ages to identify children with PUVs at increased risk of progression to ESKD and dialysis. Predictive factors reported in the literature include nadir creatinine in the first year of life, which seems to be the most reliable marker, ultrasonographic kidney volume, and urinary markers, such as albuminuria, tubular markers, and new urinary molecules (16–22).

Although PUV is a well-known disease with a relevant impact on long-term renal prognosis, there is a lack of solid literature on predictors of CKD development. We therefore decided to perform a systematic review with meta-analysis analyzing the prognostic role of creatinine nadir during the first year of life in patients with PUVs.

## Methods

### Study design

We systematically reviewed studies reporting on nadir creatinine as a predictive factor of renal outcomes in PUVs. We considered all studies that refer to the long-term kidney function outcome in patients with PUVs and the possible role of nadir creatinine as a predictor of CKD and ESKD. Our study conforms to the Preferred Reporting Items for Systematic Reviews and Meta-Analysis (PRISMA) guidelines (23, 24).

### Studies identification

We searched PubMed and Cochrane Library to include citations published from January 2008 to June 2022 without any limitations (last research on October 1, 2022), with the assistance of a medical librarian. Details of the search terms and combinations are reported in the supplementary material. Furthermore, we manually reviewed the cited references of the selected studies to identify additional potentially relevant studies.

### Studies selection

After the initial search, two investigators (NBP and DM) independently screened the identified titles and abstracts to exclude studies based on design (case reports, reviews and systematic reviews, meta-analyses, animal models, or editorials), method/intervention, or patient populations (patient > 18 years). Observational studies, both prospective and retrospective, and randomized controlled trials with more than 10 eligible patients were included.

The full text of each remaining article was manually reviewed by two investigators (NBP and DM) who were not blinded to the journal name, study authors, or institution. We excluded abstracts without full peer-reviewed publications and unavailable articles, as it was impossible to determine the clinical outcome of interest.

We selected all original research studies that include the following:

• patients aged 0–18 years who received a neonatal or antenatal diagnosis of PUV and underwent PUV correction, regardless of the type of intervention;
• collection of first year nadir creatinine data, defined as the lowest creatinine level during the first year of life of patients; and
• nadir creatinine being considered a possible predictor of CKD or ESKD development after PUV correction.

### Data collection

We abstracted the following data from each eligible study: study setting and design, journal, country, time period, population, nadir creatinine threshold in the first year, time of follow-up, incidence of CKD and ESKD, and other prognostic factors of long-term kidney function.

For relevant studies, when the required data were not presented or were unclear, we either excluded the article in its entirety or used the data only for outcomes that were clearly specified.

### Quality assessment

We assigned a quality measure to each included study using the 14-item National Institutes of Health Quality Assessment Tool for Observational Cohort and Cross-Sectional Studies (Supplementary Table S1).

Using this rating system, two investigators (RS and NBP) rated each study as either poor, fair, or good. Then, we calculated the percentage of overall agreement between the two independent reviewers’ assessments of study quality. As for the data collection procedure, disagreements between different investigators about study quality were solved through consensus with a third investigator.

### Data analysis

Six articles included suitable relative risk (RR) measures (HRs or ORs) to be included in a meta-analysis. As these two RR measures are asymmetric, all analyses were carried out on the logarithmic scale, that is, by referring to the log HRs and log ORs, although results will be reported on the original scale. A homogeneity test based on the Q statistic was performed to evaluate the between-study heterogeneity, which is summarized using the *I*^2^ index. Where significant at the 0.01 level, the summary effect, with a corresponding 95% confidence interval, was obtained from a random-effects model. A cumulative meta-analysis was carried out to assess the stability of the summary estimate. Meta-regression was used to account for possible factors of heterogeneity. Publication bias was assessed by an asymmetry of funnel plots. All analyses were carried out using the numerical computing environment R version 4.1.2 (2021-11-01) (25).

### Outcomes

Our primary endpoint was to assess nadir creatinine in the first year of life as a prognostic factor for developing CKD and ESKD. Our secondary endpoint was the identification of a cutoff of nadir creatinine and its power as prognostic factor and the identification of other prognostic factors for developing CKD and ESKD in the population considered.

## Results

**Figure 1:**
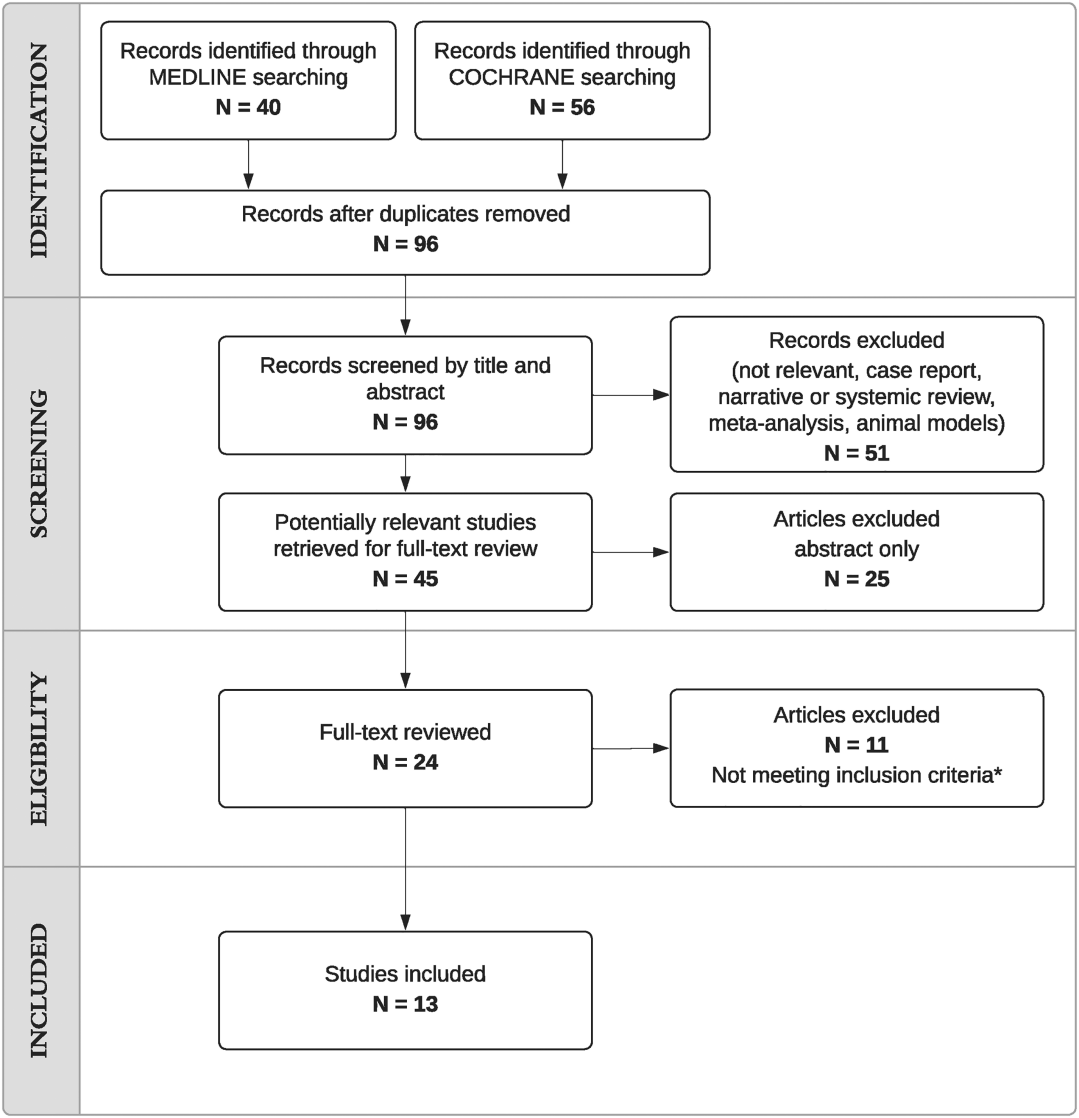
Flow chart of the study selection process.

We identified 96 potentially relevant publications for screening (Figure 1). After screening the titles and abstracts, we identified 24 studies for full-text review. Following a full-text review and the manual search of the articles included in the reference lists, we identified 13 eligible studies meeting our inclusion criteria, which were included in the review and are presented in Table 1 (26–38).

**Table 1:** Studies included in the systematic review and meta-analysis.

	Year of publication	Journal	Country	Type of study	Number of centers involved
Ansari et al.	2010	*Journal of Pediatric Urology*	India	Retrospective cohort study	Monocentric
Assefa et al.	2021	*Research and Reports in Urology*	Ethiopia	Retrospective cohort study	Monocentric
Bilgutay et al.	2015	*Journal of Pediatric Urology*	United States	Retrospective cohort study	Multicentric
Coleman et al.	2015	*Journal of Pediatric Urology*	United Kingdom	Retrospective cohort study	Monocentric
Coquillette et al.	2019	*Journal of Perinatology*	United States	Retrospective cohort study	Monocentric
DeFoor et al.	2008	*Journal of Urology*	United States	Retrospective cohort study	Monocentric
Kumar et al.	2021	*Pediatric Nephrology*	India	Retrospective cohort study	Monocentric
Lemmens et al.	2014	*Maternal-Fetal Neonatal Medicine*	Belgium	Retrospective cohort study	Monocentric
Massaguer et al.	2022	*Cirugia Pediatrica*	Spain	Retrospective cohort study	Monocentric
McLeod et al.	2019	*Pediatrics*	United States	Retrospective cohort study	Multicentric
Sarhan et al.	2011	*Journal of Urology*	Egypt	Retrospective cohort study	Monocentric
Vasconcelos et al.	2018	*Pediatric Nephrology*	Brazil	Retrospective cohort study	Monocentric
Wu et al.	2022	*Pediatric Nephrology*	USA	Retrospective cohort study	Monocentric

All the studies were based on retrospective cohort data; two studies were multicentric (28, 35), while the remaining were monocentric. The study period differed among the studies, with a range between 1975 and 2020. Overall, the 13 eligible studies included 1,731 children, for an average follow-up time of 5.5 years (range 3.5–7.0 years). A prenatal diagnosis of PUV was performed in 49.9% of patients (range 13%–100%), and these data were available for eight studies (26, 28, 31, 33, 35–38). Table 2 presents the population characteristics of the considered studies.

**Table 2:** Population characteristics of studies included in the systematic review and meta-analysis.

	Time period considered	Population			CKD prevalence (%)	ESKD prevalence (%)	Intervention for PUV correction	Prevalence of prenatal diagnosis (%)
		Inclusion criteria	*N*	Average follow-up (years)				
Ansari et al.	1992–2008	Newborns with PUVs	260	7.2	30.40	11.90	Ablation of valves	47
Assefa et al.	2015–2019	Newborns with PUVs	70	3	52.90	2.90	Endoscopic ablation and temporary vesicostomy	NA
Bilgutay et al.	2006–2014	Newborns with PUVs	104	2.4	20.20	8.60	Transurethral resection, vesicostomy, foley placement, bilateral ureterocutaneous ureterostomies	42.30
Coleman et al.	1993–2004	Newborns with PUVs	96	9.4	30.20	9.40	Primary valve ablation	NA
Coquillette et al.	2005–2014	Newborns with PUVs	34	4.5	50	15	Catheter or surgical intervention	NA
DeFoor et al.	1975–2005	Newborns with PUVs	119	7.2	NA	12.60	NA	50
Kumar et al.	1992–2015	Newborns with PUVs	270	5.5	NA	12.80	Endoscopic ablation	NA
Lemmens et al.	2001–2011	Newborns with PUVs	39	2	19	8	Bladder decompression and urethral valve ablation	74
Massaguer et al.	2010–2020	Newborns with PUVs	70	7.5	21	NA	NA	NA
McLeod et al.	1995–2005	Newborns with PUVs	274	6.3	NA	15.30	NA	63.50
Sarhan et al.	1987–2004	Newborns with PUVs	120	3.6	36.50	15	Primary valve ablation	13
Vasconcelos et al.	1970–2015	Newborns with PUVs	173	6.9	56	31	NA	35.80
Wu et al.	2003–2020	Newborns with PUVs	102	6.6	63	21	Primary valve ablation before 1 year	73.50

CKD, chronic kidney disease; PUV, posterior urethral valve; NA, not available.

The overall incidence of CKD was 37.9%, ranging 19.0%–63.0%, while the incidence of ESKD was 13.6%, ranging 8%–31%. In seven studies, newborns underwent endoscopic ablation of the urethral valves. Of these, two performed a temporary vesicostomy, and in other four studies, the type of intervention was not described (31, 34, 35, 37). Bilgutay et al. considered all the patients treated with at least one among transurethral resection of the PUV, vesicostomy, Foley placement, or bilateral cutaneous ureterostomy (28). Coquillette et al. considered newborns treated with a catheter or surgical urinary tract decompression (30).

Nadir creatinine was a significant prognostic factor for the development of CKD and ESKD in all the considered studies. The cutoff of nadir creatinine considered was between 0.7 and 2.7 mg/dL, with an average value of 1 mg/dL in the first year of life. A multivariable statistical analysis was performed in 11 studies, while Kaplan–Meier (30) and univariate (33) analyses were performed in the remaining two. The variables considered for the multivariate analysis differed among studies, and they are reported in Table 3.

**Table 3:** Main results of studies included in the systematic review and meta-analysis.

	Statistical test	Multivariate analysis		Nadir creatinine cutoff (mg/dl)	Statistical analysis results for CKD			Statistical analysis results for ESKD			Other significant prognostic factors identified through multivariate analysis
		Variables involved	N		*p*	OR	HR	*p*	OR	HR	
Ansari et al.	Multivariate statistical analysis	Severe bladder dysfunction, bilateral high-grade VUR, nadir creatinine in the first year	3	1	NA	NA	NA	0.0001	23.79	NA	Severe bladder dysfunction
Assefa et al.	Multivariate statistical analysis	Pre- and postoperative proteinuria, hypertension, delay between development of vesicostomy and ablation, nadir creatinine in the first year	5	0.8	0.001	6.914	NA	NA	NA	NA	Delay of intervention, proteinuria
Bilgutay et al.	Multivariate statistical analysis	NA	NA	2.7	0.0001	NA	NA	0.0001	NA	NA	VUR, echography abnormalities, baseline creatinine, prenatal diagnosis, prematurity
Coleman et al.	Multivariate statistical analysis	Age at diagnosis, pop-off mechanism, recurrent UTI, abnormal antenatal ultrasound, oligohydramnios, nadir creatinine in the first year	6	0.85	0.001	48.998	NA	NA	NA	NA	No
Coquillette et al.	Kaplan–Meier curve	NA	NA	1	NA	NA	NA	0.001	NA	NA	Invasive ventilation
DeFoor et al.	Multivariate statistical analysis	VUR, bladder dysfunction, nadir creatinine in the first year	3	1	NA	NA	NA	0.0001	71	NA	Bladder dysfunction
Kumar et al.	Multivariate statistical analysis	Polyuria, recurrent UTI, age <1 year, polydipsia, high-grade VUR, nadir creatinine in the first year	6	1	NA	NA	NA	0.001	NA	13.2	Severe bladder dysfunction, recurrent UTI, polyuria
Lemmens et al.	Mann–Whitney *U*-test	NA	NA	1	0.0007	1.5	NA	NA	NA	NA	No
Massaguer et al.	Multivariate statistical analysis	Nadir of urea, multiple bladder diverticula, obstructive megaureter, nadir creatinine in the first year	4	0.7	0.003	14.9	NA	NA	NA	NA	No
McLeod et al.	Multivariate statistical analysis	VUR, anticholinergic treatment, nadir creatinine in the first year	3	1	NA	NA	NA	0.001	NA	173.25	No
Sarhan et al.	Multivariate statistical analysis	Serum creatinine and creatinine clearance, renal parenchymal echogenicity, nadir creatinine in the first year	4	1	0.001	7.02	NA	NA	NA	NA	No
Vasconcelos et al.	Multivariate statistical analysis	Hypertension, baseline creatinine, proteinuria, nadir creatinine in the first year	4	0.7	0.001	NA	1.26	0.001	NA	1.31	Hypertension, baseline creatinine, proteinuria
Wu et al.	Multivariate statistical analysis	VUR, age of ablation, weight at birth, gestational age, nadir creatinine in the first year	5	0.8	0.02	15.88	NA	0.001	311.53	NA	Creatinine at 6 weeks after surgery, baseline creatinine

CKD, chronic kidney disease; ESKD, end-stage kidney disease; UTI, urinary tract infections; NA, not available.

Five studies analyzed the association between nadir creatinine and the development of CKD (27, 29, 33, 34, 36), five studies analyzed the association between nadir creatinine and the development of ESKD (26, 30, 31, 32, 35), and remaining three studies analyzed the association between nadir creatinine and the development of CKD and ESKD separately (28, 37, 38).

Eleven studies performed a multivariable analysis to screen potential confounders with the common result that nadir creatinine was an independent factor for the development of CKD. Other prognostic factors identified were proteinuria (27, 37), mild-to-severe bladder dysfunction (26, 31, 32), baseline creatinine (28, 37, 38), hypertension (37), recurrent urinary tract infection (UTI), polyuria (32), delay of intervention (27), high-grade vesico-ureteral reflux (VUR), prenatal diagnosis, renal echography abnormality, prematurity (28), creatinine at 6 weeks from the surgical ablation (38), and mechanical ventilation (30).

### Meta-analysis

**Figure 2:**
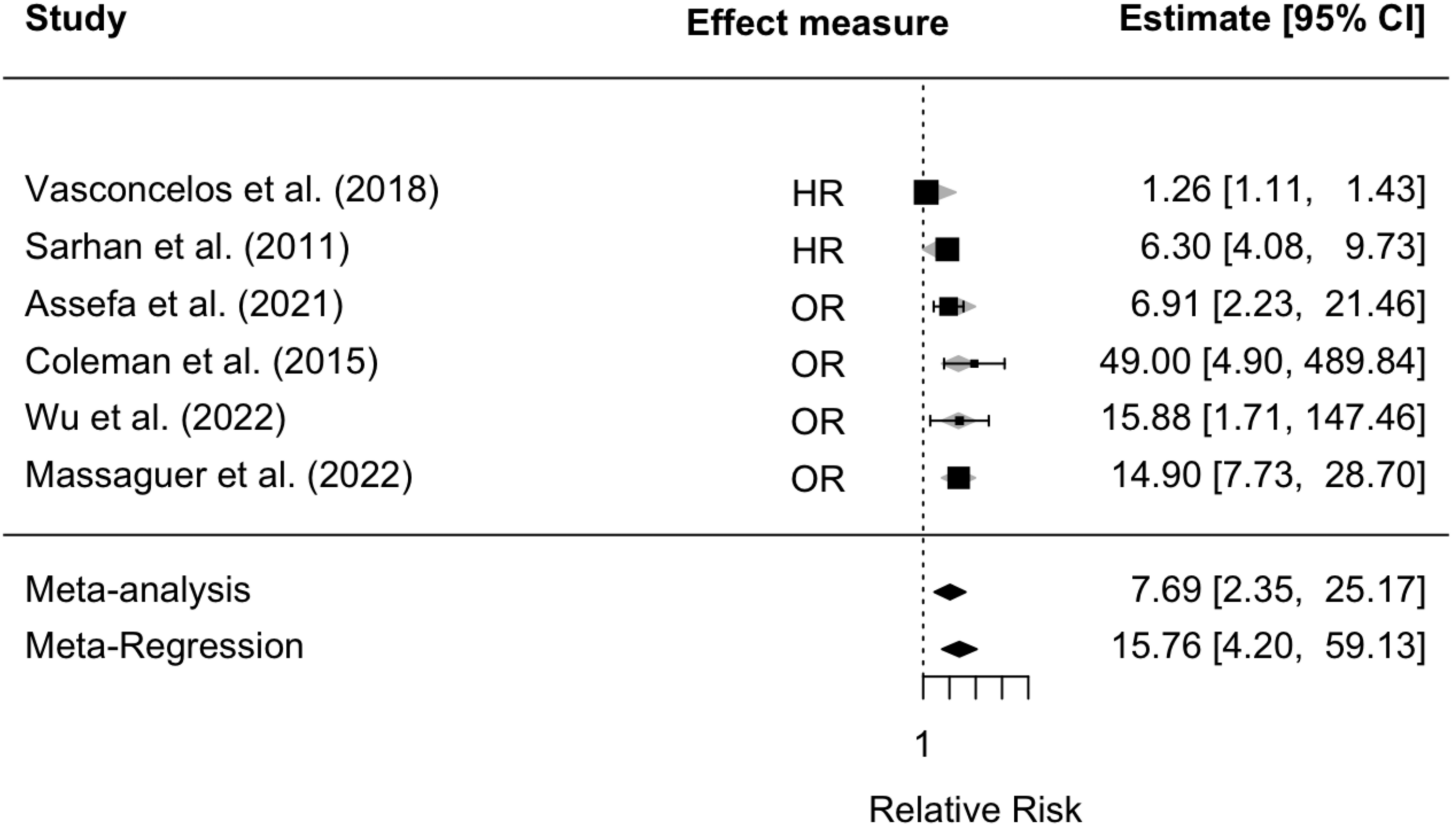
Forest plot for meta-analysis and meta-regression, accounting for the type of effect measure for the relative risk of developing CKD in patients with VUPs. CKD, chronic kidney disease; VUR, vesico-ureteral reflux.

**Figure 3:**
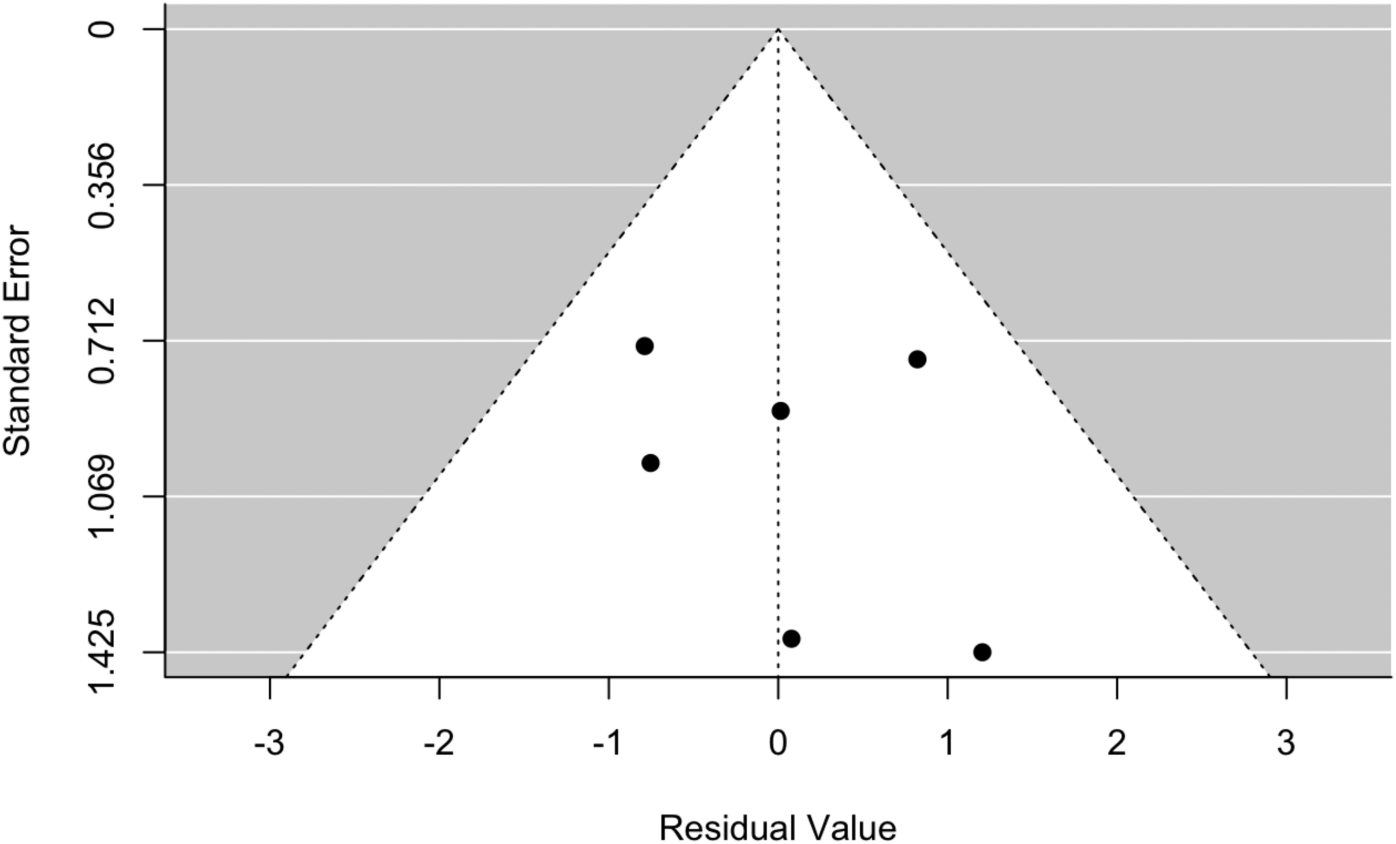
Funnel plot for meta-analysis and meta-regression, accounting for the type of effect measure, for the relative risk of developing CKD in patients with VUPs. CKD, chronic kidney disease.

Figure 2 reports the forest plot that summarizes the results of the random-effects meta-analysis carried out on the selected six studies for the CKD endpoint. It was impossible to perform a meta-analysis for the relative risk of ESKD due to the lack of data from the selected studies. In all studies, nadir creatinine was revealed to be a significant prognostic factor for the development of CKD in patients affected by PUVs (Figure 2). A summary measure of 7.7 was estimated for the RR (RR = 7.69; 95% CI: 2.35–25.17, *I*^2^ = 92.20%, *P* < 0.001), which appeared to be stable in the cumulative meta-analysis. The funnel plot showed no publication bias (Figure 3). However, the very large values of the I2 index, supported by highly significant Q statistics, highlighted, as we may have expected, a substantial heterogeneity among the studies. Accounting for the different cutoffs used through a random-effects meta-regression did not succeed in explaining this heterogeneity, although the type of effect measure (HR or RR) seems to be relevant.

## Discussion

Nadir creatinine is considered a predictor of CKD and ESKD in children affected by PUVs who underwent surgical correction, although strong literature about its reliability is still lacking (19, 39, 40).

We conducted a systematic review and meta-analysis of the risk of CKD and ESKD development based on the nadir creatinine measured in patients affected by PUVs. A previous systematic review in 2012 analyzed renal and bladder dysfunction after endoscopic treatment of LUTO, identifying nadir creatinine as the only predictive factor for the development of CKD and ESKD; however, considered studies contained heterogeneous patients and data, follow-up time, and control groups and lacked standardized reporting (14). Moreover, no nadir creatinine cutoff was identified as a significant factor in identifying patients who are at higher risk of developing CKD. Our systematic review seeks to address this literature gap. However, the clear majority of data resulted from retrospective and monocentric studies with intrinsic biases.

The most relevant result of our review is that nadir creatinine in the first year of life appears to be a reliable predictor of declining renal function in all the selected studies. The cutoff values ranged from 0.7 to 2.7 mg/dL, although most studies considered 1 mg/dL as a reference. A further important factor is that the statistical analysis, performed by multivariable analysis in most of the studies, led to highly significant results (average *p*-value of 0.005 for the development of CKD and average *p*-value of 0.0006 for ESKD). We estimated that, after about 5 years of follow-up, patients with a nadir creatinine above the threshold present a risk of developing CKD, which is 7.7 times higher than that of the other patients. This is the first study that provides a summary estimate for this type of risk. However, the meta-analysis and meta-regression suffer from the very high heterogeneity of the studies, both in terms of effect measures used (HRs and ORs) and the questionable precision of the corresponding estimates listed in the three studies.

An important topic is to stratify the risk of CKD development according to its staging. In fact, the management of children with kidney impairment depends on the stage of CKD, and the definition of a single nadir creatinine cutoff for all CKD stages is not sufficient. Wu et al. were the first to propose different cutoffs of creatinine for individual risk stratification of CKD severity; however, these data should be confirmed in further studies (40).

Moreover, nadir creatinine could be used in association with other prognostic factors to define a score for the stratification of the risk of chronic renal impairment. Coleman et al. suggested a PUV score composed of two variables: nadir creatinine and creatinine velocity, which can be calculated using simple linear regression applied to serum creatinine values during the first 5 days following bladder drainage, expressed in μmol/L/day (41). This is a new but not so diffused score, which should be validated on a larger population.

Other possible prognostic factors include echographic parameters, urinary and biochemical markers, prenatal parameters, and proteomic data. Klaus et al. concluded that the best postnatal predictor for progression to CKD was nadir serum creatinine after valve ablation, while the values of other urinary markers beyond microalbuminuria were poor, and novel postnatal biomarkers should be further investigated (42, 17). The final validation of the 12PUV signature is ongoing in the first clinical proteomics study in prenatal medicine. These fetal biomarkers showed promising results but require more extensive validation before being applied in clinical practice (43). Some genetic alterations are also related to a worse prognosis among patients with PUVs, with emphasis on renin–angiotensin system (RAS) polymorphisms, particularly those affecting the angiotensin-converting enzyme (ACE) and type II angiotensin receptor genes 1 and 2 (AGTR1 and AGTR2) (44, 45).

According to our results, bladder dysfunction and baseline creatinine are the second most frequent prognostic factor for the development of kidney impairment, identified as significant by three studies each through multivariable analysis, but the role and value of these prognostic factors are out of the scope of this paper.

### Limitations

Our results must be interpreted in the context of some limitations. Despite a rigorous search strategy and manually reviewed references in an endeavor to capture all the available data, the presence in literature of other studies not considered in the present review may not be excluded with absolute certainty. The systematic review of the literature was performed on two research systems (Medline and Cochrane Library); however, no studies of interest were identified in Cochrane Library. Moreover, the retrospective design of the studies analyzed and the lack of quality data in several fields of interest limit the evidence provided by most studies. Some articles reported missing information, and most of them were monocentric. Moreover, studies present HRs or ORs to estimate the relative risk of the development of CKD in patients with a certain cutoff of nadir creatinine. CKD is not a rare event in patients with PUVs, with an incidence of about 30% considering a mean follow-up time of only 5.5 years. Both the meta-analysis and meta-regression suffer from the very high heterogeneity of the studies, both in terms of the effect measures used (HRs and ORs) and the questionable precision of the corresponding estimates listed in three out of the six studies considered. Indeed, in these three studies, the estimated effect measures with their corresponding confidence intervals pinpoint a possible separation problem (46) that invalidates all estimation and inference. This aspect could be fixed by using a pooled analysis of all individual data retrieved from the different studies we analyzed.

## Conclusions

Nadir creatinine is currently the best prognostic factor of long-term renal function in patients affected by PUVs. A value above the cutoff of 1 mg/dl should be considered at high risk for the development of CKD and ESKD in this population. Further studies are needed to define different nadir creatinine cutoffs for better stratification of the different CKD stages and the development of reliable scores, which include the association of several variables (i.e., bladder dysfunction and proteinuria).

## Data Availability

The original contributions presented in the study are included in the article/**Supplementary Material**, further inquiries can be directed to the corresponding author.
