# Supplementary material for: Nadir creatinine as a predictor of renal outcomes in PUVs: A systematic review and meta-analysis
Source: Front Pediatr. 2023 Mar 15;11:1085143. doi: 10.3389/fped.2023.1085143 (PMC10050680; doi:10.3389/fped.2023.1085143)
Supplement: Supplementary file 1 [file Table1.docx]

**Electronic Supplementary Material**

**Search strategy**

**TABLE S1: Quality Assessment Tool for Observational Cohort and Cross-Sectional Studies**

**TABLE S2: Methodological quality of included studies to evaluate the outcome**

**TABLE S3: Quality rating of included studies using the National Institutes of Health Quality Assessment Tool for Observational Cohort and Cross-Sectional Studies**

**Search Strategy MEDLINE/PubMed**

1. **Nadir creatinine**

AND

1. **Posterior urethral valves**

(Posterior urethral valves OR LUTO OR PUV)

AND

1. **Renal function outcome**

(CKD OR chronic kidney disease OR dialysis OR renal failure OR ESRD OR end stage renal disease OR renal outcome OR prognosis)

Time limit: 01/01/2008-14/10/2022

**Search Strategy The Cochrane Library**

1# Nadir creatinine [Mesh] AND

2# renal function [Mesh] OR

3# posterior urethral valve [Mesh]

**TABLE S1: Quality Assessment Tool for Observational Cohort and Cross-Sectional Studies**

(https://www.nhlbi.nih.gov/health-topics/study-quality-assessment-tools, last accessed October 15, 2022)

| **Criteria** | **Yes** | **No** | **Other**  **(CD, NR, NA)*** |
| --- | --- | --- | --- |
| 1. Was the research question or objective in this paper clearly stated? |  |  |  |
| 2. Was the study population clearly specified and defined? |  |  |  |
| 3. Did the literature search strategy use a comprehensive,  systematic approach? |  |  |  |
| 4. Were all the subjects selected or recruited from the same or similar populations (including the same time period)?  Were inclusion and exclusion criteria for being in the study  prespecified and applied uniformly to all participants? |  |  |  |
| 5. Was a sample size justification, power description, or variance and effect estimates provided? |  |  |  |
| 6. For the analyses in this paper, were the exposure(s) of interest measured prior to the outcome(s) being measured? |  |  |  |
| 7. Was the timeframe sufficient so that one could reasonably expect to see an association between exposure and outcome if it existed? |  |  |  |
| 8. For exposures that can vary in amount or level, did the study examine different levels of the exposure as related to the outcome (e.g., categories of exposure, or exposure measured as continuous variable)? |  |  |  |
| 9. Were the exposure measures (independent variables) clearly defined, valid, reliable, and implemented consistently across all study participants? |  |  |  |
| 10. Was the exposure(s) assessed more than once over time? |  |  |  |
| 11. Were the outcome measures (dependent variables) clearly defined, valid, reliable, and implemented consistently across all study participants? |  |  |  |
| 12. Were the outcome assessors blinded to the exposure status of participants? |  |  |  |
| 13. Was loss to follow-up after baseline 20% or less? |  |  |  |
| 14. Were key potential confounding variables measured and adjusted statistically for their impact on the relationship between exposure(s) and outcome(s)? |  |  |  |

**Quality Rating (Good, Fair, or Poor) (see guidance)**

*CD, cannot determine; NA, not applicable; NR, not reported

**TABLE S2: Methodological quality of included studies to evaluate the outcome - Final results based on consensus between investigator**

| **Studies** | | **1. Objective** | **2. Population**  **definition** | **3. Participant rate** | **4. Selection criteria** | **5. Sample size** | **6. Exposure** | **7. Timeframe** | **8. Levels of exposure** | **9. Exposures measures** | **10. Repeated exposure** | **11. Outcome measures** | **12. Blinding** | **13. Loss to follow-up** | **14. Confounding**  **variables** |
| --- | --- | --- | --- | --- | --- | --- | --- | --- | --- | --- | --- | --- | --- | --- | --- |
| Ansari et al. | 2010 | Yes | Yes | NA | Yes | No | Yes | Yes | NA | Yes | NA | Yes | NA | Yes | Yes |
| Assefa et al. | 2021 | Yes | Yes | Yes | Yes | Yes | Yes | No | Yes | Yes | NA | Yes | NA | No | Yes |
| Bilgutay et al. | 2015 | Yes | Yes | NA | No | No | Yes | NR | NA | Yes | NA | Yes | NA | No | Yes |
| Coleman et al. | 2015 | Yes | Yes | NA | Yes | No | Yes | Yes | Yes | Yes | NA | Yes | NA | No | Yes |
| Coquillette et al. | 2019 | Yes | Yes | NA | Yes | No | Yes | Yes | Yes | Yes | NA | Yes | NA | No | No |
| DeFoor et al. | 2008 | Yes | Yes | NA | Yes | No | Yes | Yes | No | Yes | NA | Yes | NA | Yes | Yes |
| Kumar et al. | 2021 | Yes | Yes | NA | Yes | No | Yes | Yes | Yes | Yes | Yes | Yes | NA | Yes | Yes |
| Lemmens et al. | 2014 | Yes | Yes | NA | Yes | No | Yes | Yes | Yes | Yes | Yes | Yes | NA | No | No |
| Massaguer et al. | 2022 | Yes | Yes | NA | Yes | No | Yes | Yes | Yes | Yes | Yes | Yes | NA | Yes | Yes |
| McLeod et al. | 2019 | Yes | Yes | NA | Yes | No | Yes | Yes | Yes | Yes | Yes | Yes | NA | No | Yes |
| Sarhan et al. | 2011 | Yes | Yes | NA | Yes | No | Yes | Yes | No | No | Yes | Yes | NA | NR | Yes |
| Vasconcelos et al. | 2018 | Yes | Yes | NA | Yes | No | Yes | Yes | Yes | Yes | Yes | Yes | NA | Yes | Yes |
| Wu et al. | 2022 | Yes | Yes | NA | Yes | No | Yes | Yes | Yes | Yes | Yes | Yes | NA | Yes | Yes |

NA: Not Available; NR: Not Reported

**TABLE S3: Quality rating of included studies using the National Institutes of Health Quality Assessment Tool for Observational Cohort and Cross-Sectional Studies**

| **Studies** | **Publication**  **year** | **Quality rating,**  **Reviewer 1** | **Quality rating,**  **Reviewer 2** | **Agreement**  **between**  **reviewers** | **Consensus**  **rating** |
| --- | --- | --- | --- | --- | --- |
| Ansari et al. | 2010 | G | F | Yes | G |
| Assefa et al. | 2021 | G | G | Yes | G |
| Bilgutay et al. | 2015 | G | G | Yes | G |
| Coleman et al. | 2015 | F | G | Yes | G |
| Coquillette et al. | 2019 | F | G | Yes | G |
| DeFoor et al. | 2008 | F | G | Yes | G |
| Kumar et al. | 2021 | G | G | Yes | G |
| Lemmens et al. | 2014 | G | G | Yes | G |
| Massaguer et al. | 2022 | G | F | Yes | G |
| McLeod et al. | 2019 | G | G | Yes | G |
| Sarhan et al. | 2011 | G | G | Yes | G |
| Vasconcelos et al. | 2018 | G | G | Yes | G |
| Wu et al. | 2022 | G | P | Yes | G |

G = Good

F = Fair

P = Poor
